# Ability of the triglyceride–glucose index and C-reactive protein-to-albumin ratio to predict severity of acute pancreatitis and 28-day mortality

**DOI:** 10.1371/journal.pone.0359089

**Published:** 2026-09-28

**Authors:** Qiuhui Tian, Yinyin Zhang, Xiaolin Zhu, Ting Luo, Qiumei Cao, Yu Liu

**Affiliations:** 1 Department of Gastroenterology, Chongqing Jiangjin District Hospital of Chinese Medicine, Chongqing, China; 2 Department of Gastroenterology, Jiangjin Hospital, Chongqing University of Chinese Medicine, Chongqing, China; University of Montenegro-Faculty of Medicine, MONTENEGRO

## Abstract

**Background:**

Severe acute pancreatitis (SAP) is characterized by rapid development, complex clinical changes, and a high mortality rate. Early identification of patients with acute pancreatitis that has the potential to become severe is important for optimizing clinical outcomes. However, at present, there is no effective prediction or evaluation tool. This study aimed to determine the ability of the triglyceride–glucose (TyG) index and C-reactive protein-to-albumin ratio (CAR) to predict the risk of progression to SAP and 28-day mortality.

**Methods:**

This retrospective cohort study included 316 patients with a diagnosis of acute pancreatitis. Information on background characteristics, including sex, age, comorbidities, etiological factors, other relevant variables, and laboratory results was collected. The primary endpoints were progression to SAP and 28-day mortality in patients with SAP. Associations of the TyG index and CAR with the risk of SAP were assessed by logistic regression. Prediction performance was evaluated by receiver-operating characteristic curve analysis and internally validated via 1,000 bootstrap resamples with calibration. Cox regression and Firth’s penalized Cox regression were used to evaluate 28-day mortality. Survival was compared between groups using the Kaplan–Meier method.

**Results:**

TyG index and CAR values were significantly higher in the SAP group than in the non-SAP group (both *p* < 0.001). After full adjustment for potential confounders, multivariate logistic regression, the TyG index (odds ratio 2.42, 95% CI 1.36–4.32, *p* = 0.003) and CAR (odds ratio 1.40, 95% CI 1.12–1.74, *p* = 0.003) were independently associated with SAP. The apparent areas under the curve for the TyG index and CAR for prediction of SAP were 0.702 and 0.793, respectively; following bootstrap internal validation, the optimism-corrected areas under the curve were 0.701 and 0.793, respectively, with optimism values below 0.05 in both cases and calibration curves demonstrating good agreement between predicted and observed probabilities. Firth’s penalized likelihood Cox regression revealed positive associations of elevated TyG index and CAR values with 28-day mortality in patients with SAP, with respective hazard ratios of 11.90 (95% CI 3.47–50.53) and 10.22 (95% CI 2.31–96.19). Kaplan–Meier survival analysis confirmed that clinical outcomes were significantly worse in patients with SAP and a TyG index ≥12.12 or CAR ≥ 3.72 (*p* < 0.001, log-rank test).

**Conclusion:**

The TyG index and CAR were identified as independent predictors of progression from acute pancreatitis to SAP. High-risk thresholds (TyG index ≥12.12 and CAR ≥ 3.72) showed good prediction performance for 28-day mortality in patients with SAP, highlighting their value for early risk stratification. Nevertheless, these cut-off values require validation in prospective multicenter cohorts.

## Introduction

Acute pancreatitis (AP) is an acute inflammatory disorder triggered by aberrant activation of pancreatic enzymes that drive autodigestion of the pancreas and adjacent tissues. Epidemiological data indicate a global annual incidence of 30–40 cases per 100,000 individuals [1]. The pathogenesis of AP is multifactorial: while 65%–70% of patients present with mild, self-limiting disease, approximately 20% progress to severe acute pancreatitis (SAP), a life-threatening subtype defined by persistent organ dysfunction, refractoriness to treatment, and poor outcomes [2]. Mortality in patients with SAP varies according to type of complication and has been reported to be 12% for sterile pancreatic necrosis, 30% for infected necrosis, and 47% in cases complicated by multiple organ failure [3]. These figures underscore the hallmarks of SAP, which are rapid progression, clinical heterogeneity, and high lethality. Early identification of patients at risk of progression to SAP, coupled with intensified monitoring and targeted interventions, remains a critical unmet need for optimization of clinical outcomes.

Current clinical tools for assessment of the severity of AP, including the Ranson, Glasgow, Acute Physiology and Chronic Health Evaluation (APACHE) II, and Computed Tomography Severity Index scores, are limited by cumbersome indicator panels, operational complexity, high costs, or long turnaround times [4]. The prediction performance of conventional laboratory markers (e.g., white blood cell count, lactate dehydrogenase, triglycerides, D-dimer, serum potassium, and calcium) has been explored [5]. However, their sensitivity and specificity in early-stage assessment remain suboptimal, highlighting the need for more accessible and responsive biomarkers to enable timely risk stratification and intervention.

The triglyceride–glucose (TyG) index is a novel composite indicator for assessment of insulin resistance [6] and has the advantages of easy acquisition, low cost, and dynamic monitoring. In recent years, it has demonstrated value for prediction of cardiovascular events and a spectrum of gastrointestinal disorders [7–9]. Integration of the TyG index into traditional risk models has been shown to substantially improve prediction performance for SAP, pointing to its potential value in evaluation of short-term AP outcomes [10]. Nevertheless, the quantitative relationship between the TyG index and the severity/prognosis of AP remains poorly defined, restricting its translation into clinical stratification protocols.

The C-reactive protein-to-albumin ratio (CAR) is a dual-purpose marker that reflects both systemic inflammatory activity and nutritional status. Research indicates that CAR outperforms both C-reactive protein and albumin when each is used alone for quantification of the burden of inflammation [11] and that it has prognostic relevance across diverse conditions, including malignancy, cardiovascular/cerebrovascular disease, and AP [12]. Kaplan et al. have reported on the value of CAR as a predictor of AP-related mortality [13], but data on its association with the severity of AP and long-term outcomes remain scarce.

This study aimed to determine the ability of the combined application of the TyG index and CAR to predict the risk of AP becoming severe and the prognosis at 28 days. Our hope was to provide a basis for assessment of the prognosis, risk stratification, and individualized treatment of AP, thereby standardizing the clinical diagnosis of SAP and its treatment and improving the long-term prognosis.

## Materials and methods

### Study design and patient cohort

A total of 384 patients diagnosed with AP who were admitted to Chongqing Jiangjin District Hospital of Chinese Medicine between January 1, 2020 and October 10, 2024 were retrospectively enrolled in this study. The research data were accessed for analysis on June 30, 2025. Eligibility criteria included age ≥ 18 years and a confirmed diagnosis of AP, which required at least two of the following three criteria to be met: AP-related gastrointestinal symptoms; serum amylase or lipase activity ≥3 times the upper reference limit; and radiological findings consistent with AP [14]. The severity of AP was evaluated according to the revised Atlanta classification as follows: mild AP (without organ dysfunction or local or systemic complications); moderately severe AP (with transient [≤48 hours] organ dysfunction and/or local complications); and SAP (accompanied by persistent [>48 hours] organ failure) [15]. For analytical purposes, patients with mild or moderately severe AP were pooled into a non-SAP group. The etiology of AP was categorized as gallstone-related, hypertriglyceridemia-related, alcohol-related, or other.

The following exclusion criteria were applied: symptom onset-to-admission interval >72 hours (n = 47), age < 18 years (n = 2), pregnancy (n = 1), concurrent malignancy (n = 5), pre-existing chronic heart/liver/kidney insufficiency (n = 8), or incomplete clinical data (n = 5). After 68 exclusions, the final study cohort consisted of 316 patients with AP. The study protocol was approved by our institutional ethics committee (approval number KY20240920). The requirement for informed consent was waived in view of the retrospective observational nature of the research.

### Data collection

Demographic, clinical, and laboratory data obtained within 24 hours of admission were extracted from the electronic medical records. Fasting venous blood samples were collected from all enrolled patients on the morning after admission (8–12 hours post-admission) using a standardized collection time between 6:00 am and 7:00 am on the first working day following admission. Demographic and clinical data included age, sex, history of AP, hypertension, type 2 diabetes mellitus (T2DM), and etiology of AP. Laboratory parameters included procalcitonin, C-reactive protein, white blood cell count, neutrophil count, lymphocyte count, platelet count, total bilirubin, alanine aminotransferase, aspartate aminotransferase, creatinine, blood urea nitrogen, uric acid, calcium, triglycerides, fasting blood glucose, and albumin. The TyG index was calculated as Ln [triglycerides (mg/dL) × fasting blood glucose (mg/dL)/2] and the CAR as C-reactive protein/albumin. Serum triglyceride, fasting glucose, C-reactive protein, and albumin were measured using the same batch of reagents for all participants. Comorbidities and clinical outcomes, including concurrent infection, pancreatic necrosis, transfer to the intensive care unit (ICU), and 28-day mortality, were documented for all patients who developed SAP. The data were collected and evaluated independently by at least two of the investigators. Multivariate regression imputation was used for variables with a missing rate below 5%; variables with a missing rate above this cut-off were omitted from the analysis. All subsequent evaluations included the complete-case dataset.

### Statistical analysis

Quantitative data that were normally distributed were compared between groups using the independent samples *t*-test, and those that were not normally distributed are expressed as the median [interquartile range] and were compared using the Mann–Whitney *U* test. Qualitative data are expressed as the number (percentage) and were compared between groups using the chi-squared test or Fisher’s exact test. The study population was divided into four groups based on quartiles (Q1-Q4) according to the TyG index value and CAR. Multivariate regression analysis was used to evaluate the association between each of these variables and SAP. Model 1 was not adjusted for any potential confounding factors; model 2 was adjusted for age, sex, comorbidities (hypertension, T2DM), and history of AP; and model 3 was adjusted further for laboratory variables. The ability of the TyG index and CAR to predict SAP was analyzed using the receiver-operating characteristic (ROC) curve and the area under the curve (AUC). Internal validation was performed using bootstrap resampling with 1,000 iterations, from which the optimism-corrected AUC was derived. Calibration curves were generated to evaluate the agreement between predicted and observed outcomes. The optimal cut-off values for the ability of the TyG index and CAR to predict 28-day mortality in patients with SAP were calculated by ROC curve analysis. A multivariate Cox proportional hazards regression model was established to assess the associations of the TyG index and CAR with the risk of 28-day mortality in patients with SAP: model 1 was not adjusted for any potential confounding factors, whereas model 2 was adjusted for age and sex. Given the limited number of events (3.67 events per variable), we refitted the multivariable model using Firth’s penalized likelihood Cox regression, a method designed to reduce small-sample bias and address separation with the aim of obtaining more stable estimates. Survival curves were plotted using the Kaplan–Meier method and compared between groups using the log-rank test. All statistical analyses were performed using R software (version 4.2.2; R Foundation for Statistical Computing, Vienna, Austria), SPSS software (version 27.0; IBM Corp., Armonk, NY, USA), and GraphPad Prism (version 10.1.2; GraphPad Software Inc., San Diego, CA, USA). All statistical analyses were two-sided, and a *p*-value of <0.05 was considered statistically significant.

## Results

### Baseline characteristics of patients with AP

A total of 384 patients diagnosed with AP were initially screened for this study. Following application of strict inclusion and exclusion criteria, 316 patients were included in the final analysis (Fig 1). The patients were stratified based on disease severity into a non-SAP group (n = 238) and a SAP group (n = 78). The baseline clinical characteristics of the two groups are summarized in Table 1. Compared with patients in the non-SAP group, those in the SAP group were older and more likely to have T2DM and to be experiencing their first episode of AP. Gallstones and hypertriglyceridemia were the most common underlying etiologies in the SAP group. Laboratory assessments revealed that procalcitonin, white blood cell count, neutrophil count, alanine aminotransferase, aspartate aminotransferase, creatinine, TyG index, and CAR values were significantly higher in the SAP group than in the non-SAP group and that the serum calcium level was significantly lower in the SAP group (all *p* < 0.05).

**Table 1:** Baseline patient characteristics according to severity of acute pancreatitis.

Characteristic	Overall (N = 316)	Non-SAP (N = 238)	SAP (N = 78)	*p*-value
**General Information**				
Sex, n (%)				0.706
Female	136 (43.0%)	101 (42.4%)	35 (44.9%)	
Male	180 (57.0%)	137 (57.6%)	43 (55.1%)	
Age	50 (40, 57)	48 (39, 55)	53 (43, 65)	0.002
History of AP, n (%)				0.013
No	156 (49.4%)	108 (45.4%)	48 (61.5%)	
Yes	160 (50.6%)	130 (54.6%)	30 (38.5%)	
Type 2 diabetes mellitus, n (%)				<0.001
No	218 (69.0%)	185 (77.7%)	33 (42.3%)	
Yes	98 (31.0%)	53 (22.3%)	45 (57.7%)	
Hypertension, n (%)				0.903
No	168 (53.2%)	127 (53.4%)	41 (52.6%)	
Yes	148 (46.8%)	111 (46.6%)	37 (47.4%)	
Causes, n (%)				<0.001
Alcoholic	97 (30.7%)	91 (38.2%)	6 (7.7%)	
Gallstones	53 (16.8%)	33 (13.9%)	20 (25.6%)	
Hypertriglyceridemia	137 (43.4%)	90 (37.8%)	47 (60.3%)	
Others	29 (9.2%)	24 (10.1%)	5 (6.4%)	
**Laboratory indicators**				
Procalcitonin (ng/mL)	0.18 (0.05, 0.45)	0.10 (0.04, 0.24)	1.09 (0.32, 5.40)	<0.001
White blood cell count (×10^9^/L)	11.7 (8.9, 14.7)	11.4 (8.3, 13.8)	12.7 (10.0, 16.2)	0.002
Neutrophilic (×10^9^/L)	9.8 (6.7, 12.3)	9.4 (6.1, 11.7)	10.7 (7.9, 13.6)	0.001
Lymphocyte (×10^9^/L)	1.40 (1.00, 1.90)	1.46 (1.07, 1.91)	1.15 (0.80, 1.80)	0.018
Platelet (×10^9^/L)	211 (160, 267)	208 (162, 258)	221 (145, 312)	0.201
Total bilirubin (μmol/L)	14 (10, 20)	13 (11, 19)	16 (10, 26)	0.111
Alanine aminotransferase (U/L)	27 (18, 43)	26 (18, 38)	32 (22, 67)	0.002
Aspartate aminotransferase (U/L)	27 (19, 47)	24 (19, 37)	45 (26, 100)	<0.001
Blood urea nitrogen (mmol/L)	4.94 (4.03, 6.32)	4.80 (3.81, 5.66)	6.20 (4.50, 8.25)	<0.001
Creatinine (μmol/L)	52 (37, 70)	50 (35, 65)	66 (50, 101)	<0.001
Uric Acid (μmol/L)	344 (265, 411)	344 (269, 404)	344 (261, 429)	0.960
Calcium (mmol/L)	2.37 (2.16, 2.56)	2.40 (2.25, 2.64)	2.15 (1.88, 2.40)	<0.001
TyG index	10.75 (9.60, 11.57)	10.53 (9.38, 11.40)	11.36 (10.07, 12.02)	<0.001
CAR	0.79 (0.14, 2.23)	0.39 (0.12, 1.51)	3.12 (1.32, 5.60)	<0.001

**Fig 1:**
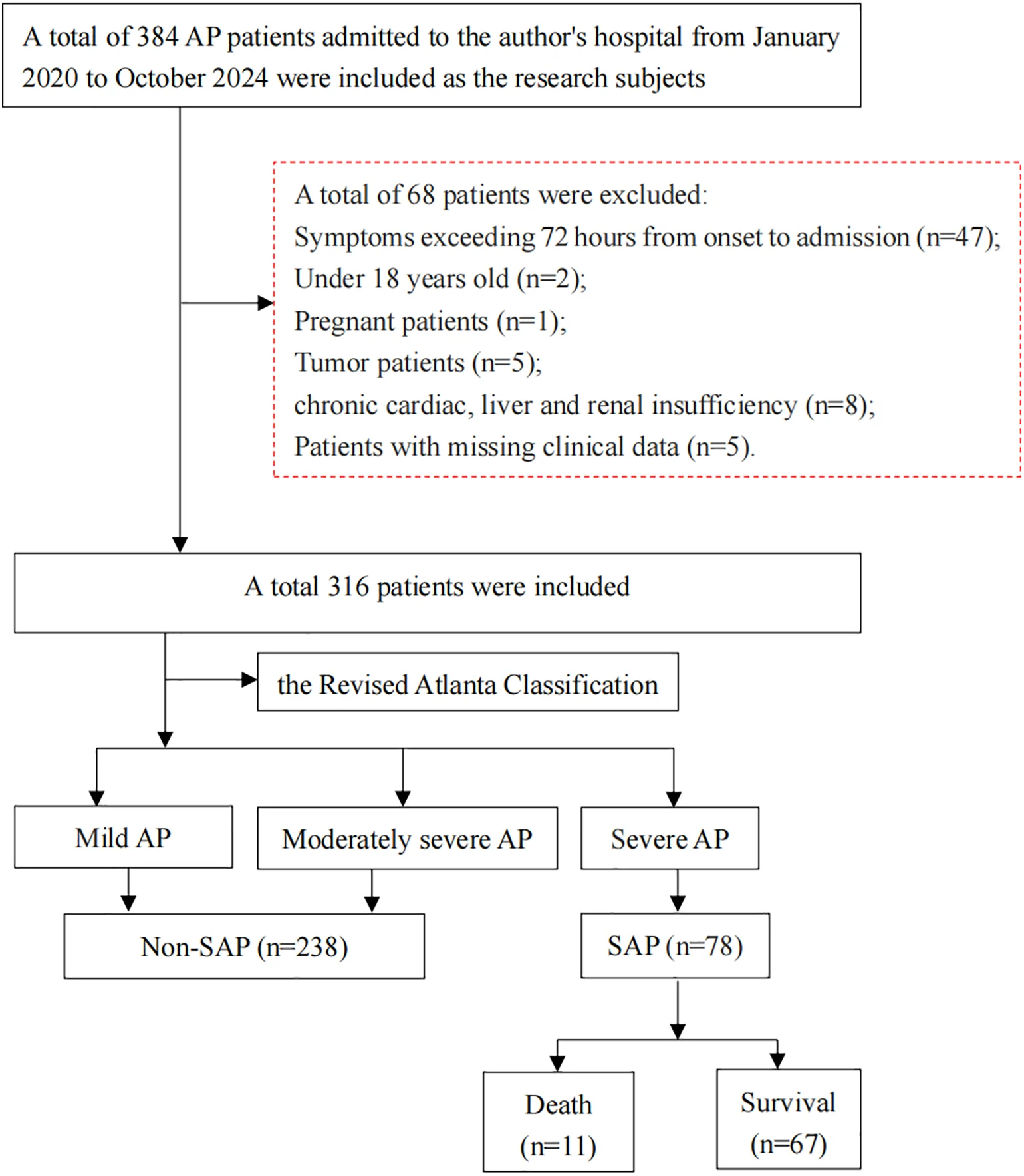
Flow chart showing the patient selection process.

### Prevalence of SAP based on the TyG index and CAR

The distribution of the prevalence of SAP across quartiles (Q1–Q4) is shown according to the TyG index and CAR in Fig 2. For the TyG index, the quartile distributions differed significantly between the SAP and non-SAP groups (*p* = 0.004). The proportion of patients in the upper quartile was higher in the SAP group than in the non-SAP group (Q4: 38.5% vs. 20.6%) and the proportion in the lower quartile was smaller in the SAP group (Q1: 14.1% vs. 28.6%). A similar pattern emerged for CAR quartiles, with marked intergroup differences (*p* < 0.001). The proportion in Q4 was substantially higher in the SAP group than in the non-SAP group (59.0% vs. 13.9%) and the proportions in Q1 and Q2 were significantly lower in the SAP group (9.0% vs. 30.3% and 7.7% vs. 30.7%, respectively). Collectively, these data demonstrate that elevated TyG index and CAR values are significantly associated with development of SAP.

**Fig 2:**
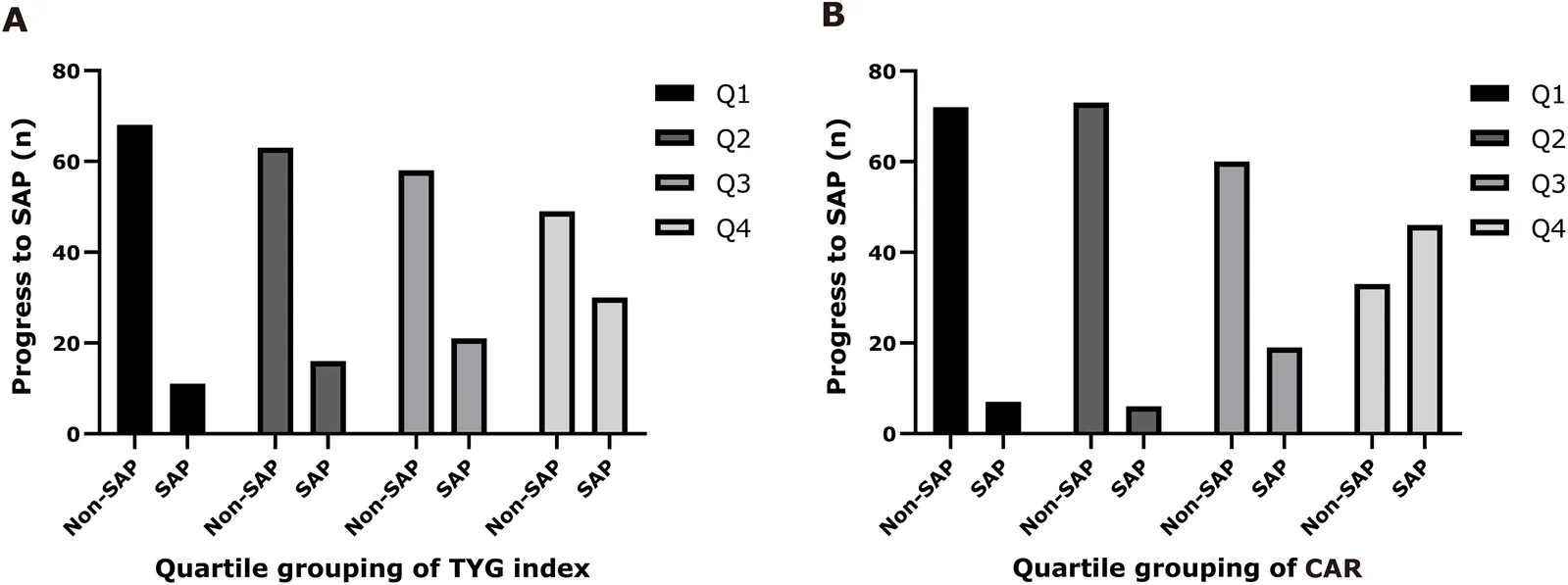
Prevalence of severe acute pancreatitis based on the TyG index and CAR. (A) TyG index. (B) CAR. CAR, C-reactive protein-to-albumin ratio; TyG, triglyceride–glucose.

### Association between the TyG index, CAR, and SAP

Table 2 shows the results of the logistic regression models examining the associations of the TyG index and CAR with the risk of SAP. In the unadjusted model 1, each 1-unit increment in the TyG index was associated with a 63% higher risk of SAP (odds ratio [OR] 1.63, 95% confidence interval [CI] 1.30–2.06, *p* < 0.001). After adjusting for demographic and clinical factors, namely, age, sex, history of AP, hypertension, T2DM, and etiology of AP (model 2), the TyG index had greater prediction performance (OR 2.16, 95% CI 1.41–3.29, *p* < 0.001). The association remained significant in model 3, which was adjusted further for inflammatory and biochemical markers (OR 2.42, 95% CI 1.36–4.32, *p* = 0.003).

**Table 2:** Findings in multivariate logistic regression models examining the associations of the TyG index and CAR with the risk of severe acute pancreatitis.

Characteristic	Model 1		Model 2		Model 3	
	OR (95% CI)	*p*-value	OR (95% CI)	*p*-value	OR (95% CI)	*p*-value
TyG (continuous)	1.63 (1.30, 2.06)	<0.001	2.16 (1.41, 3.29)	<0.001	2.42 (1.36, 4.32)	0.003
TyG						
Q1 [7.43,9.60)	—		—		—	
Q2 [9.60,10.80)	1.57 (0.68, 3.64)	0.293	3.16 (1.11, 9.04)	0.031	3.69 (1.00, 13.66)	0.051
Q3 [10.80,11.60)	2.24 (1.00, 5.03)	0.051	3.47 (1.02, 11.82)	0.047	2.92 (0.57, 14.89)	0.197
Q4 [11.60,13.60]	3.78 (1.73, 8.28)	<0.001	6.77 (1.68, 27.24)	0.007	7.33 (1.14, 47.08)	0.036
P for trend		<0.001		0.009		0.038
CAR (continuous)	1.82 (1.55, 2.14)	<0.001	1.86 (1.54, 2.25)	<0.001	1.40 (1.12, 1.74)	0.003
CAR						
Q1 [0.007,0.143)	—		—		—	
Q2 [0.143,0.787)	0.85 (0.27, 2.64)	0.772	0.75 (0.21, 2.66)	0.653	0.76 (0.20, 2.89)	0.682
Q3 [0.787,2.230)	3.26 (1.28, 8.27)	0.013	3.42 (1.20, 9.71)	0.021	2.75 (0.86, 8.74)	0.087
Q4 [2.230,14.100]	14.34 (5.86, 35.11)	<0.001	18.07 (6.24, 52.36)	<0.001	5.01 (1.25, 19.99)	0.023
P for trend		<0.001		<0.001		0.005

Model 1: no covariates were adjusted. Model 2: adjusted for sex, age, history of AP, hypertension, and type 2 diabetes mellitus. Model 3: adjusted for sex, age, history of AP, hypertension, type 2 diabetes mellitus, etiology, procalcitonin, white blood cell count, neutrophilic, lymphocyte, platelet, total bilirubin, alanine aminotransferase, aspartate aminotransferase, blood urea nitrogen, creatinine, uric acid, calcium.

When stratified by TyG index quartile (Q1–Q4, with Q1 as the reference), model 2 showed a significantly elevated risk of SAP in Q2 (OR 3.16, 95% CI 1.11–9.04, *p* = 0.031), Q3 (OR 3.47, 95% CI 1.02–11.82, *p* = 0.047), and Q4 (OR 6.77, 95% CI 1.68–27.24, *p* = 0.007). In model 3, Q4 remained the highest risk group (OR 7.33, 95% CI 1.14–47.08, *p* = 0.036). A significant dose–response relationship was observed across all models (*p* for trend <0.05).

The continuous CAR variable also showed a significant positive association with the risk of SAP. In model 1, each 1-unit increment in the CAR increased the risk of SAP by 82% (OR 1.82, 95% CI 1.55–2.14, *p* < 0.001). This association persisted in models 2 and 3.

For CAR quartiles (using Q1 as the reference), the risk of SAP was significantly higher in Q3 and Q4 in models 1 and 2. In model 3, only Q4 retained statistical significance (OR 5.01, *p* = 0.023). Trend tests confirmed a significant dose–response pattern between CAR and the risk of SAP across all models (*p* < 0.05).

Building on the above analyses, we generated ROC curves to assess the ability of the TyG index and CAR to predict SAP (Fig 3). When treated as continuous variables, the TyG index and CAR had baseline AUCs of 0.702 and 0.793, respectively, for prediction of SAP. Internal validation was performed using the bootstrap resampling method with 1,000 iterations (Table 3). After correction, the optimism value was below 0.05 for all models, indicating minimal overfitting and satisfactory predictive robustness. Calibration curves for the univariate TyG index and CAR models are presented in Fig 4. The smoothed calibration curves (blue solid lines) for both models closely aligned with the 45° ideal reference line (red dashed line), indicating good agreement between predicted probabilities and observed outcomes and supporting satisfactory model calibration. These findings suggest that the prediction performance of the TyG index and CAR was not substantially overestimated and may have good generalizability.

**Table 3:** Bootstrap internal validation of the TyG index-based and CAR-based logistic regression models for predicting severe acute pancreatitis in patients with severe acute pancreatitis.

TyG index				CAR			
Model	Apparent	Corrected	Optimism	Model	Apparent	Corrected	Optimism
Model 1	0.702	0.701	0.001	Model 1	0.793	0.793	−0.000
Model 2	0.808	0.791	0.017	Model 2	0.863	0.849	0.014
Model 3	0.934	0.890	0.044	Model 3	0.914	0.863	0.050

**Fig 3:**
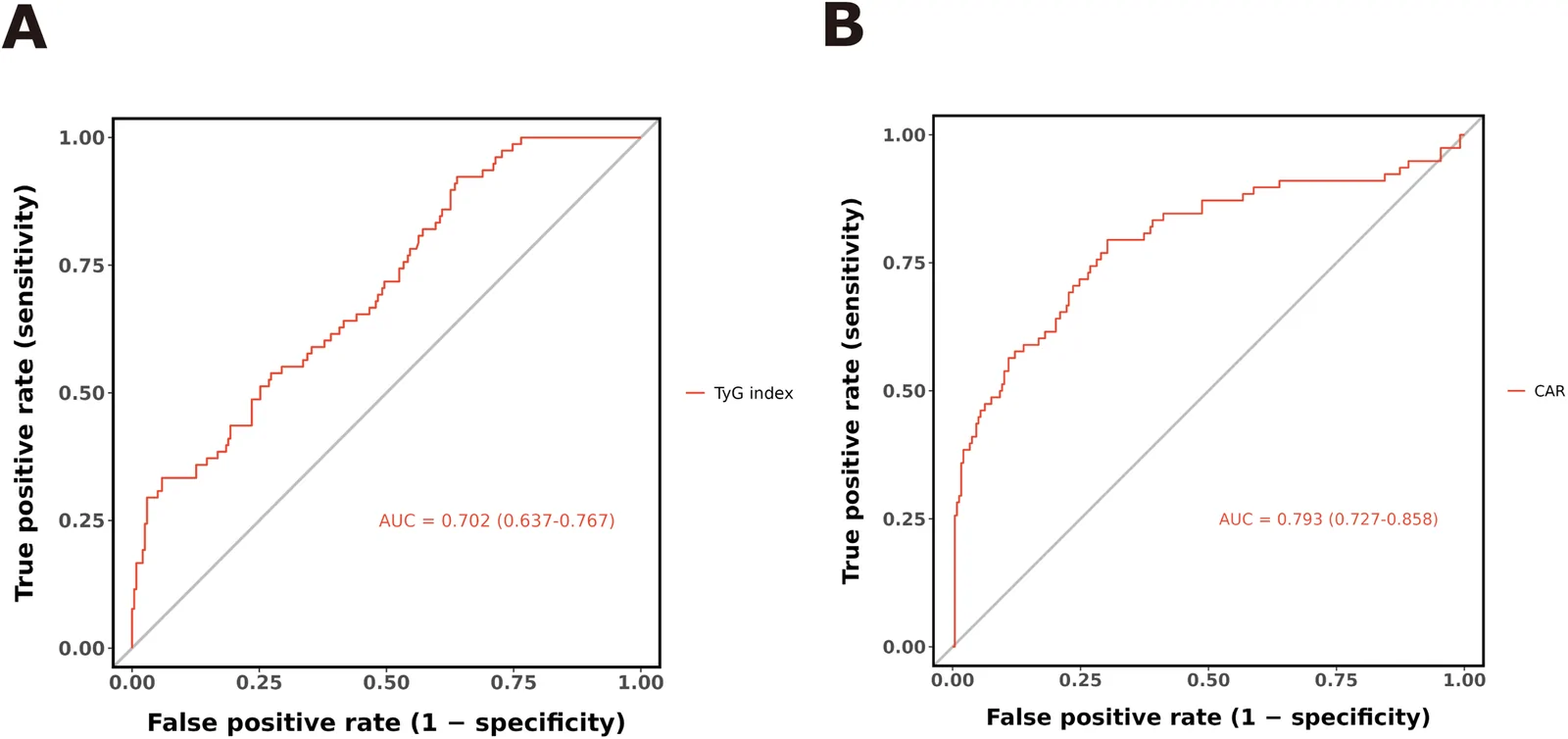
Ability of the TyG index and CAR to predict progression to severe acute pancreatitis. (A) TyG index and (B) CAR serving as continuous variables. CAR, C-reactive protein-to-albumin ratio; TyG, triglyceride–glucose.

**Fig 4:**
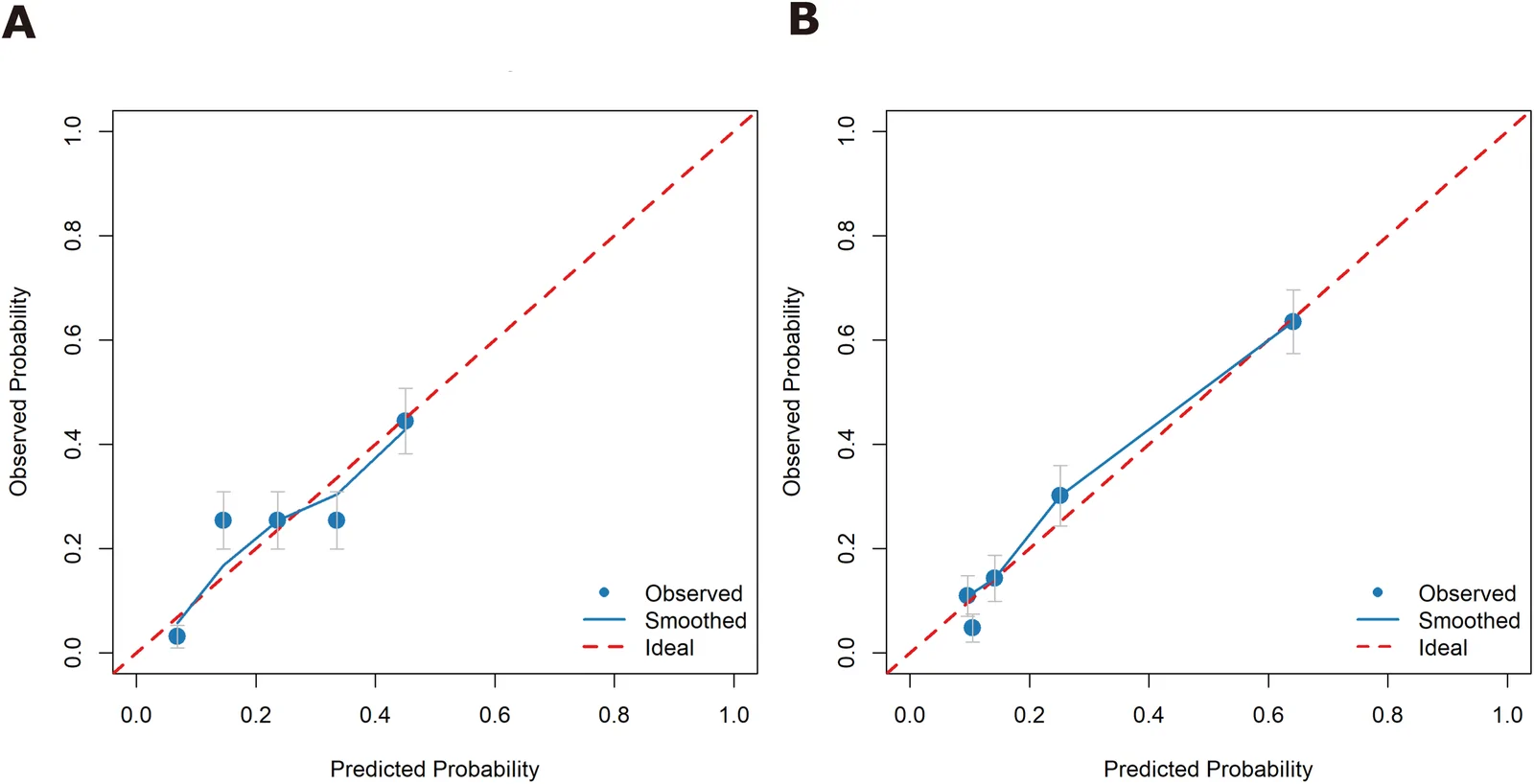
Calibration curves for logistic regression models of the TyG index (A) and CAR (B) as predictors of severe acute pancreatitis. CAR, C-reactive protein-to-albumin ratio; TyG, triglyceride–glucose.

### Ability of the TyG index and CAR to predict 28-day mortality in patients with SAP

The prediction performance of the TyG index and CAR for 28-day mortality in patients with SAP was assessed using ROC analysis (Table 4). Optimal cut-off values were identified to be 12.12 for the TyG index and 3.72 for the CAR. Both indicators were dichotomized based on these thresholds, and their ROC curves for predicting SAP mortality are presented in Fig 5. When analyzed as continuous variables, the AUCs for the TyG index and CAR were 0.826 and 0.856, respectively; after dichotomization at the optimal cut-offs (TyG index ≥12.12; CAR ≥ 3.72), the corresponding AUCs were 0.811 and 0.768. The combination of the two biomarkers yielded an AUC of 0.891 (continuous) and 0.866 (dichotomized), which was higher than that of either marker alone. These findings indicate that integrating both biomarkers improves the ability to predict 28-day mortality in patients with SAP.

**Table 4:** Exploratory TyG index and CAR thresholds for prediction of 28‑day mortality in patients with acute pancreatitis.

Characteristic	AUC	Cut-off	Youden	Sensitivity	Specificity	Accuracy	Precision
TyG index	0.826	12.12	0.62	72.7%	89.6%	87.2%	53.3%
CAR	0.856	3.72	0.63	100.0%	62.7%	67.9%	30.6%

**Fig 5:**
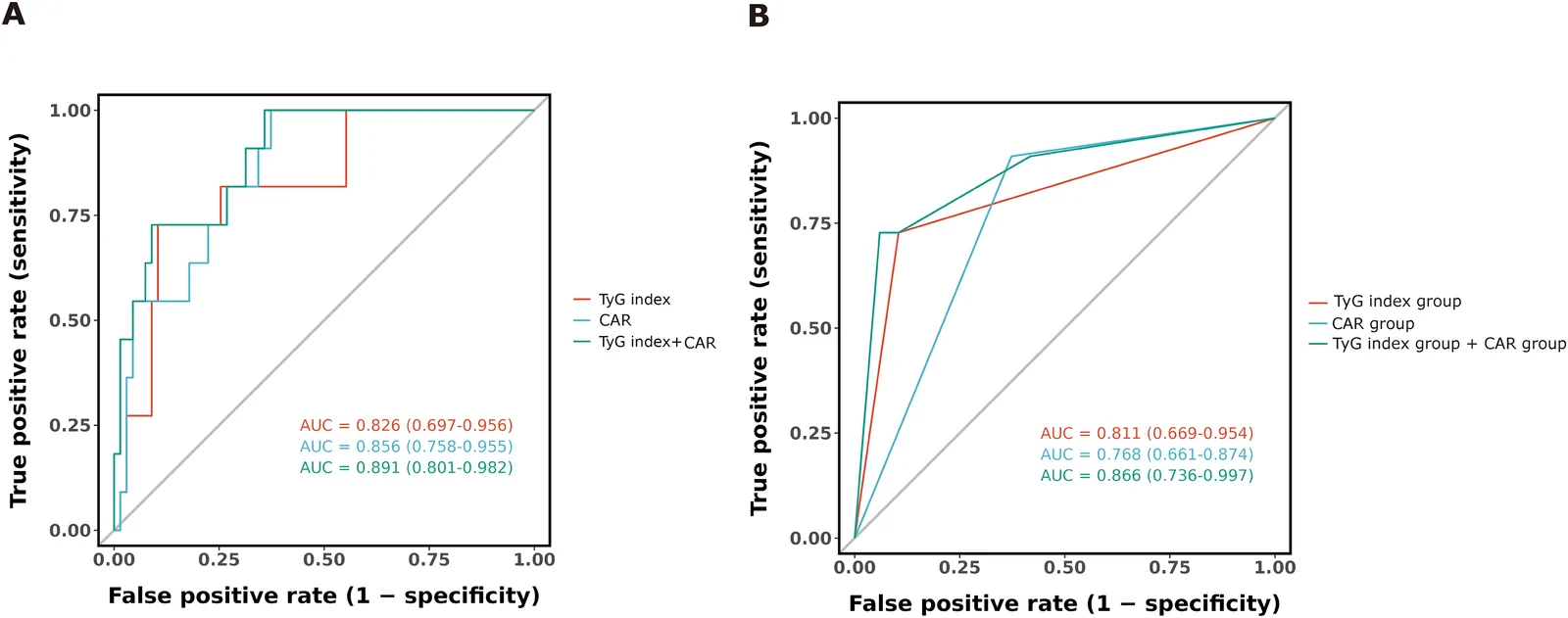
Ability of the TyG index and CAR to predict 28-day mortality in patients with severe acute pancreatitis. (A) The TyG index and CAR serving as a continuous variable. (B) The TyG index and CAR serving as a binary categorical variable. CAR, C-reactive protein-to-albumin ratio; TyG, triglyceride–glucose.

### Baseline characteristics of patients with SAP stratified by TyG index and CAR

As shown in Table 5, patients with SAP and a high TyG index (≥12.12) had a higher prevalence of hypertriglyceridemia as the etiology, an elevated procalcitonin level, a lower lymphocyte count, an increased incidence of pancreatic necrosis, more concurrent infections, and more transfers to the ICU, as well as significantly greater 28-day mortality compared to those with a lower TyG index (<12.12; *p* < 0.05). Similarly, patients with a high CAR (≥3.72) were more likely to have hypertriglyceridemia and elevated procalcitonin, white blood cell count, neutrophil, blood urea nitrogen, and uric acid levels along with increased ICU admission rates and 28-day mortality relative to the low CAR group (<3.72; *p* < 0.05).

**Table 5:** Demographic, clinical, and laboratory data for patients with severe acute pancreatitis according to TyG index and CAR values.

Characteristic	TyG index group		*p*-value	CAR group		*p*-value
	< 12.12 (N = 63)	≥ 12.12 (N = 15)		< 3.72 (N = 43)	≥ 3.72 (N = 35)	
**General Information**						
Sex, n (%)			0.463			0.216
Female	27 (42.9%)	8 (53.3%)		22 (51.2%)	13 (37.1%)	
Male	36 (57.1%)	7 (46.7%)		21 (48.8%)	22 (62.9%)	
Age	55 (43, 68)	50 (39, 56)	0.207	55 (48, 65)	50 (37, 69)	0.124
History of AP, n (%)			0.188			0.472
No	41 (65.1%)	7 (46.7%)		28 (65.1%)	20 (57.1%)	
Yes	22 (34.9%)	8 (53.3%)		15 (34.9%)	15 (42.9%)	
Type 2 diabetes mellitus, n (%)			0.774			0.929
No	31 (49.2%)	8 (53.3%)		18 (41.9%)	15 (42.9%)	
Yes	32 (50.8%)	7 (46.7%)		25 (58.1%)	20 (57.1%)	
Hypertension, n (%)			0.097			0.274
No	36 (57.1%)	5 (33.3%)		25 (58.1%)	16 (45.7%)	
Yes	27 (42.9%)	10 (66.7%)		18 (41.9%)	19 (54.3%)	
Causes, n (%)			0.004			0.005
Alcoholic	6 (9.5%)	0 (0.0%)		6 (14.0%)	0 (0.0%)	
Gallstones	20 (31.7%)	0 (0.0%)		15 (34.9%)	5 (14.3%)	
Hypertriglyceridemia	32 (50.8%)	15 (100.0%)		20 (46.5%)	27 (77.1%)	
Others	5 (7.9%)	0 (0.0%)		2 (4.7%)	3 (8.6%)	
**Laboratory indicators**						
Procalcitonin (ng/mL)	1 (0, 4)	12 (1, 24)	0.003	0 (0, 1)	5 (2, 15)	<0.001
White blood cell count (×10^9^/L)	12.4 (10.1, 15.9)	14.9 (8.8, 17.3)	0.985	11.3 (9.3, 15.3)	15.5 (11.1, 20.2)	0.002
Neutrophilic (×10^9^/L)	10.4 (8.0, 13.1)	12.8 (7.4, 15.6)	0.756	9.2 (7.8, 12.6)	12.8 (9.2, 15.6)	0.010
Lymphocyte (×10^9^/L)	1.28 (0.89, 2.00)	0.98 (0.60, 1.13)	0.017	1.17 (0.83, 2.00)	1.13 (0.70, 1.71)	0.778
Platelet (×10^9^/L)	227 (145, 331)	191 (109, 269)	0.385	198 (145, 314)	234 (141, 311)	0.821
Total bilirubin (μmol/L)	17 (10, 26)	14 (9, 28)	0.766	14 (10, 25)	17 (10, 27)	0.488
Alanine aminotransferase (U/L)	33 (22, 67)	31 (18, 81)	0.577	35 (25, 79)	28 (18, 67)	0.379
Aspartate aminotransferase (U/L)	41 (24, 103)	57 (32, 86)	0.568	43 (25, 100)	47 (27, 115)	0.790
Blood urea nitrogen (mmol/L)	6 (5, 8)	7 (4, 17)	0.526	6 (4, 7)	7 (5, 11)	0.047
Creatinine (μmol/L)	70 (50, 101)	60 (38, 171)	0.581	63 (50, 95)	66 (44, 114)	0.563
Uric Acid (μmol/L)	342 (232, 414)	371 (262, 550)	0.342	299 (220, 367)	408 (275, 526)	0.002
Calcium (mmol/L)	2.17 (1.93, 2.40)	2.13 (1.13, 2.54)	0.756	2.18 (1.96, 2.40)	2.11 (1.72, 2.45)	0.605
**Complications**						
Pancreatic necrosis, n (%)			0.015			0.703
No	47 (74.6%)	6 (40.0%)		30 (69.8%)	23 (65.7%)	
Yes	16 (25.4%)	9 (60.0%)		13 (30.2%)	12 (34.3%)	
Concurrent infection, n (%)			0.013			0.191
No	35 (55.6%)	3 (20.0%)		36 (83.7%)	25 (71.4%)	
Yes	28 (44.4%)	12 (80.0%)		7 (16.3%)	10 (28.6%)	
Admitted to the ICU, n (%)			0.003			<0.001
No	54 (85.7%)	7 (46.7%)		33 (76.7%)	5 (14.3%)	
Yes	9 (14.3%)	8 (53.3%)		10 (23.3%)	30 (85.7%)	
Outcome, n (%)			<0.001			0.002
Death	3 (4.8%)	8 (53.3%)		1 (2.3%)	10 (28.6%)	
Survival	60 (95.2%)	7 (46.7%)		42 (97.7%)	25 (71.4%)	

### Association of the TyG index and CAR with 28-day mortality in patients with SAP

Cox regression analysis in the unadjusted model 1 revealed that both a high TyG index and high CAR were significantly associated with an increased risk of mortality, with HRs of 14.57 (95% CI 3.85–55.10; *p* < 0.001) and 14.10 (95% CI 1.80–110.22; *p* = 0.012), respectively. In model 2, which adjusted for age and sex, the mortality risk remained significantly elevated (*p* < 0.05). When the multivariable model was refitted using Firth’s penalized likelihood Cox regression (model 3), the associations were further quantified, with hazard ratios of 11.90 (95% CI 3.47–50.53) for the TyG index and 10.22 (95% CI 2.31–96.19) for CAR (Table 6). Kaplan–Meier analysis confirmed that patients with a TyG index of ≥12.12 and a CAR of ≥3.72 had significantly poorer survival (*p* < 0.001; Fig 6A,6B).

**Table 6:** Association of TyG index and CAR (binary) with 28-day mortality in patients with severe acute pancreatitis: Cox and Firth’s penalized Cox regression analyses.

	Cox regression analysis				Firth’s penalized Cox regression	
Characteristic	Model 1		Model 2		Model 3	
	HR (95% CI)	*p*-value	HR (95% CI)	*p*-value	HR (95% CI)	*p*-value
TyG index group						
< 12.12	—		—		—	
≥ 12.12	14.57 (3.85, 55.10)	<0.001	14.00 (3.51, 55.86)	<0.001	11.90 (3.47, 50.53)	<0.001
CAR group						
< 3.72	—		—		—	
≥ 3.72	14.10(1.80, 110.22)	0.012	14.40 (1.80, 115.48)	0.012	10.22 (2.31, 96.19)	0.001

Model 1: no covariates were adjusted. Model 2: adjusted for age and sex. Model 3: Firth’s penalized Cox regression, adjusted for age and sex.

**Fig 6:**
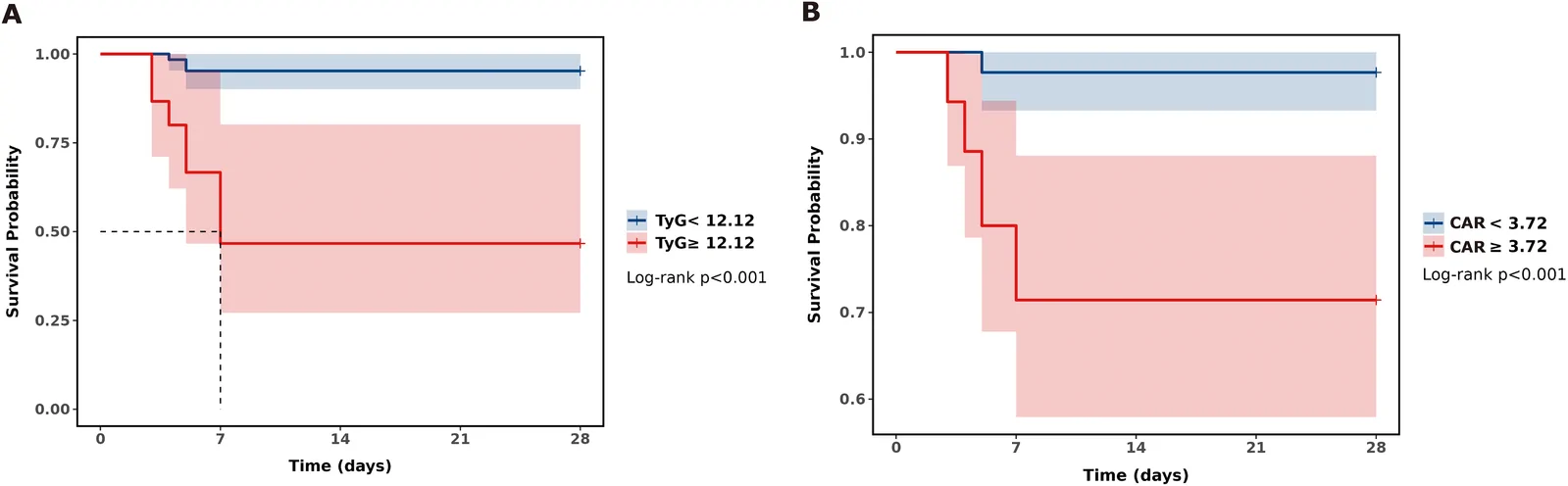
Association of the TyG index (A) and CAR (B) with 28-day mortality in patients with severe acute pancreatitis. CAR, C-reactive protein-to-albumin ratio; TyG, triglyceride–glucose.

## Discussion

AP is a common digestive emergency and is characterized by rapid progression and a high risk of severe complications [16]. Patient outcomes depend on early accurate assessment of disease severity and timely intervention. While mild AP can often be managed effectively by conservative treatment, including fluid resuscitation, analgesia, and nutritional support, severe cases frequently require intensive care and potentially mechanical ventilation or surgery [17]. Therefore, early identification of high-risk patients is essential to guide intensive monitoring and treatment, thereby improving the prognosis. This study evaluated the ability of the TyG index and CAR to predict both progression to SAP and 28-day mortality in patients with SAP. Its key findings are as follows. First, patients who progressed to SAP were older and more likely to have T2DM and to be initially diagnosed to have AP, with gallstones and hypertriglyceridemia as the predominant etiologies. They also had significantly higher levels of inflammatory markers, more severe hepatic and renal impairment, and an elevated TyG index and CAR (*p* < 0.05). Second, multivariable logistic regression showed that both the TyG index and CAR were independently associated with SAP; bootstrap validation provided corrected AUCs of 0.701 and 0.793, respectively, with optimism values under 0.05, suggesting minimal overfitting. Third, in multivariable Cox regression analysis, an elevated TyG index and CAR emerged were independently associated with 28-day mortality 28-day mortality in patients with SAP. This association remained robust after applying Firth’s penalized likelihood correction (p ≤0.001). Fourth, Kaplan–Meier analysis revealed significantly worse survival among patients with a TyG index ≥12.12 and CAR ≥ 3.72 (*p* < 0.001, log-rank test).

This study identified the systemic inflammatory response, multi-organ dysfunction, metabolic disturbances, and inflammation–nutrition imbalance as characteristic pathological features in patients with SAP. Consistent with previous cross-sectional studies [18], patients with SAP tended to be older than those with mild AP. There is a bidirectional relationship between diabetes and pancreatitis in which diabetes elevates the risk of pancreatitis while hyperglycemia may aggravate systemic organ failure in patients with AP [19]. Conversely, SAP is a known risk factor for new-onset diabetes, particularly pancreatogenic diabetes [20]. Epidemiological data from China indicate that hypertriglyceridemia-induced AP accounts for 10%–30% of all cases of AP [21]. Hypertriglyceridemia-induced AP is more likely to progress to SAP than other etiological subtypes and is associated with higher rates of organ failure, systemic infection, pancreatic necrosis, and ICU admission [22]. Furthermore, persistent mechanical obstruction by gallstones, if not relieved by endoscopy or surgery, promotes continuous activation of enzymes and exacerbation of inflammation, contributing to development of SAP [23]. In this study, most patients with SAP were newly diagnosed, suggesting that limited disease awareness may result in delayed consultation and missed opportunities for early intervention. Although current guidelines recommend an initial assessment of severity at admission based on systemic inflammatory response syndrome or prolonged organ failure (>48 hours) [24], these criteria are more applicable in advanced stages involving infected necrosis or organ dysfunction, and have limited early predictive value. Therefore, the development of accurate early prediction tools is critical to guide timely clinical intervention, mitigate disease progression, reduce complications, and ultimately improve patient outcomes.

Both the TyG index and CAR were independently associated with SAP. The TyG index, a composite biomarker derived from fasting triglyceride and glucose levels [25], has been shown to outperform traditional measures of insulin resistance such as the hyperinsulinemic–euglycemic clamp [26] and HOMA-IR (homeostasis model assessment of insulin resistance) [27] in identifying metabolic dysfunction. It is also closely associated with the pathogenesis and progression of insulin resistance-related conditions, including metabolic syndrome, T2DM, cardiovascular disease, and non-alcoholic fatty liver disease [28]. Glucose and lipid metabolism play critical roles in the pathophysiology of AP. The onset of AP involves pancreatic edema, ischemia, and necrosis, leading to islet cell injury, impaired insulin secretion, and sustained hyperglycemia [29, 30]. Hyperglycemia not only reflects endocrine dysfunction but also amplifies inflammation by promoting production of inflammatory mediators and cellular infiltration [31]. Hypertriglyceridemia elevates serum free fatty acids, intensifies pancreatic lipolysis, and generates toxic unsaturated fatty acids that impair the mitochondria, stimulate release of pro-inflammatory cytokines, and damage ductal epithelium [32, 33]. Furthermore, high triglyceride levels increase blood viscosity, contributing to microvascular occlusion and impaired pancreatic microcirculation [34]. These mechanisms collectively provide a pathological basis for the robust association between an elevated TyG index and the risk of SAP.

CAR is a composite indicator reflecting both systemic inflammation and nutritional status. Although widely used as a marker of cancer-associated systemic inflammation [35], its clinical relevance in AP remains unclear. C-reactive protein, an acute-phase protein synthesized by the liver, increases during inflammation and serves as a non-specific inflammatory biomarker. Its value in stratifying the severity of AP has been investigated [4]. Conversely, albumin, a negative acute-phase reactant, decreases under inflammatory conditions [36]. A previous study reported that patients with AP and serum albumin levels of <25 g/L during hospitalization had a 48.8-fold increase in risk of developing severe AP [37]. Therefore, elevated CAR may signal worsening inflammatory and nutritional status. The evidence suggests that CAR more accurately reflects inflammatory states than either CRP or albumin alone [38].

These findings suggest that the TyG index and CAR may be associated with clinical outcomes in patients with SAP and that elevations in these variables are significantly associated with increased 28-day mortality. Patients with a TyG index ≥12.12 and a CAR ≥ 3.72 had a significantly worse prognosis (*p* < 0.001, log-rank test). Wei Y et al. [39] reported a TyG index cut-off value of 8.40 with an AUC of 0.784, sensitivity of 75.0%, and specificity of 73.2%. In contrast, our study identified a higher TyG index threshold of 12.12 with an AUC of 0.826, sensitivity of 72.7%, and specificity of 89.6%. Kaplan et al. [13] observed that each unit increase in CAR was associated with a 1.52-fold increase in risk of mortality, with a CAR > 16.28 predicting mortality at 92.1% sensitivity and 58.0% specificity. A lower CAR cut-off of 3.72 yielded an AUC of 0.856 with sensitivity of 100.0% and specificity of 62.7%. The observed association between CAR and clinical outcomes may reflect its dual role as a marker of both inflammatory activity and depletion of nutritional reserve, both of which are interrelated pathways that may influence disease progression. Our findings suggest that the TyG index and CAR could serve as useful biomarkers for risk stratification in patients with AP, although optimal thresholds may differ across populations and warrant further investigation.

In this exploratory cohort, the combination of the TyG index and CAR showed an AUC of 0.891 for 28-day mortality, suggesting potential incremental value beyond that when these biomarkers are used individually. This improvement in performance may be attributed to the association between insulin resistance (as reflected by the TyG index) and metabolic dysregulation with elevated pro-inflammatory factors, including nuclear factor-κB, tumor necrosis factor-α, islet amyloid polypeptide, and interleukin-6 [2]. These cytokines drive a potent pro-inflammatory immune response that amplifies initial pancreatic damage and propagates systemic inflammation via lymphatic and circulatory pathways, potentially leading to systemic inflammatory response syndrome [2] and multi-organ dysfunction through positive feedback between cytokine storms and programmed immune cell death [40]. Such processes may worsen the inflammation–nutrition imbalance captured by CAR, creating a vicious cycle of metabolic disturbance and uncontrolled inflammation that collectively aggravates the severity of AP and contributes to poor outcomes.

This study has several limitations. First, its single-center retrospective design may have introduced selection bias, considering that the study population was from a tertiary hospital in Southwest China and might not fully represent the broader community spectrum of SAP. Second, the generalizability of our findings is constrained by their regional and ethnic composition; metabolic profiles, etiological patterns, and healthcare accessibility vary across populations, so the proposed cut-offs (TyG index ≥12.12, CAR ≥ 3.72) require external validation in diverse cohorts. Third, the Cox model was limited by a low event count (n = 11, approximately 3.67 events per variable). Although we applied Firth’s correction to reduce small-sample bias, the results need confirmation in larger cohorts. Sample size constraints also limited covariate adjustment, meaning that the possibility of residual confounding could not be excluded. Fourth, unmeasured confounders, including clinical severity scores (e.g., BISAP, APACHE II, SOFA) and early interventions, could not be accounted for. Multicenter prospective studies with larger sample sizes are needed to validate and extend our findings.

## Conclusion

Both the TyG index and CAR were independently associated with the severity and prognosis of AP, with high-risk thresholds (TyG index ≥12.12, CAR ≥ 3.72) linked to poorer outcomes in patients with SAP. These routinely available biomarkers are non-invasive and cost-effective. Nevertheless, the proposed cut-offs are exploratory and derived from a single-center retrospective study. Therefore, prospective multicenter validation is needed to confirm their clinical usefulness.
